# Optimal blending of multiple independent prediction models

**DOI:** 10.3389/frai.2023.1144886

**Published:** 2023-02-24

**Authors:** Peter Taraba

**Affiliations:** Independent Researcher, Fort Lauderdale, FL, United States

**Keywords:** blending of independent models, normal distributions, machine learning with counts, Gaussians, going wider

## Abstract

We derive blending coefficients for the optimal blend of multiple independent prediction models with normal (Gaussian) distribution as well as the variance of the final blend. We also provide lower and upper bound estimation for the final variance and we compare these results with machine learning with counts, where only binary information (feature says yes or no only) is used for every feature and the majority of features agreeing together make the decision.

## Introduction

Participants of the Netflix competition used model blending heavily—refer to, for example Töscher et al. (2009), Amatriain (2013), Xiang and Yang (2009), Coscrato et al. (2020), Koren (2009), Jahrer et al. (2010), and Bothos et al. (2011). Ensemble modeling (blending) was popular not only in Netflix competition but is used also on other machine learning problems such as image processing, for example for CIFAR-10 dataset, refer to Abouelnaga et al. (2016) and Bruno et al. (2022), for the MNIST dataset refer to Ciresan et al. (2011). Ensemble modeling is used also in many other fields, for example, refer to Schuhen et al. (2012) and Ardabili et al. (2020). In this study, we derive blending coefficients based on variances of different models with only the assumption of model independence. While the formula for the final variance of the blended model and its coefficients is already derived in Kay (1993) without a proof (Equations 6.7 and 6.8 in chapter 6.4), we provide proofs both for the formula for blending coefficients and the variance of the combined model as well as the lower and upper bound estimate for the final variance based on the minimal and maximal variance of all the combined models. We also compare these results with machine learning with counts, where only binary information is used from the features to make the decision, in the last section and show very similar conclusions.

Let ŷ_*k,j*_ be a prediction of model *k* ∈ [1, *N*] for element *j* ∈ [1, *M*], where *N* is the number of different independent models and *M* is the number of measurements we have:


$$\begin{array}{l} {ŷ}_{k,j}=y_{j}+r_{k,j}, \end{array}$$

where *y*_*j*_ is an expected prediction and *r*_*k,j*_ is a random variable with normal distribution $R_{k}\sim N\left(0,\sigma_{k}^{2}\right)$, which has a zero average (the expected value of the variable is 0). In this study, we derive optimal blending coefficients α_*k*_ such that the blended prediction ŷ_*B*_ is optimal:


$$\begin{array}{l} {ŷ}_{B,j}=\overset{N}{\underset{k=1}{\sum }}\alpha_{k}{ŷ}_{k,j}=y_{j}\overset{N}{\underset{k=1}{\sum }}\alpha_{k}+\overset{N}{\underset{k=1}{\sum }}\alpha_{k}r_{k,j}=y_{j}+\overset{N}{\underset{k=1}{\sum }}\alpha_{k}r_{k,j} \end{array}$$

with minimum variance $\sigma_{B}^{2}$, where $\overset{N}{\underset{k=1}{\sum }}\alpha_{k}=1$.

## Blending two independent models

Here we present two independent models


$$\begin{array}{l} {ŷ}_{1,j}=y_{j}+r_{1,j}\\ {ŷ}_{2,j}=y_{j}+r_{2,j}, \end{array}$$

where $R_{1}\sim N\left(0,\sigma_{1}^{2}\right)$ and $R_{2}\sim N\left(0,\sigma_{2}^{2}\right)$. We derive $\hat{\alpha }\in \left[0,1\right]$ for which we get the optimal blending model


$$\begin{array}{l} {ŷ}_{B,j}=\alpha \left(y_{j}+r_{1,j}\right)+\left(1-\alpha \right)\left(y_{j}+r_{2,j}\right)=y_{j}+\alpha r_{1,j}\\ +\left(1-\alpha \right)r_{2,j}. \end{array}$$

It is well-known fact that a random variable combining two random variables α*R*_1_ + (1 − α)*R*_2_, where $R_{1}\sim N\left(0,\sigma_{1}^{2}\right)$, $R_{2}\sim N\left(0,\sigma_{2}^{2}\right)$ and *R*_1_ and *R*_2_ are independent, has a normal distribution $N\left(0,\sigma_{B}^{2}\right)$, where $\sigma_{B}^{2}=\alpha^{2}\sigma_{1}^{2}+{\left(1-\alpha \right)}^{2}\sigma_{2}^{2}$. For the mean we get:


$$\begin{array}{l} E\left(Y_{B}\right)=\frac{1}{M}\overset{M}{\underset{j=1}{\sum }}\left(\alpha r_{1,j}+\left(1-\alpha \right)r_{2,j}\right)\\ =\alpha E\left(R_{1}\right)+\left(1-\alpha \right)E\left(R_{2}\right)=0 \end{array}$$

and for the variance we get:


$$\begin{array}{l} E\left(Y_{B}^{2}\right)=\frac{1}{M}\overset{M}{\underset{j=1}{\sum }}{\left(\alpha r_{1,j}+\left(1-\alpha \right)r_{2,j}\right)}^{2}=\\ =\alpha^{2}\frac{1}{M}\overset{M}{\underset{j=1}{\sum }}r_{1,j}^{2}+2\alpha \left(1-\alpha \right)\frac{1}{M}\overset{M}{\underset{j=1}{\sum }}r_{1,j}r_{2,j}+{\left(1-\alpha \right)}^{2}\frac{1}{M}\overset{M}{\underset{j=1}{\sum }}r_{2,j}^{2}. \end{array}$$

Finally as *R*_1_ and *R*_2_ are independent (covariance $\frac{1}{M}\underset{j=1}{\sum }r_{1,j}r_{2,j}=0$ is zero), we can write:


$$\begin{array}{l} \sigma_{B}^{2}=E\left(Y_{B}^{2}\right)=\alpha^{2}\sigma_{1}^{2}+{\left(1-\alpha \right)}^{2}\sigma_{2}^{2}. \end{array}$$

To find the optimal (we are looking for minimal value and function is convex with one minimum as we have only α^0^, α^1^, and α^2^ dependencies—quadratic function and $\sigma_{1}^{2}+\sigma_{2}^{2}>0$) blending parameter, we compute where a partial derivative of the new variance of the blended model is zero:


$$\begin{array}{l} \frac{\partial \sigma_{B}^{2}}{\partial \alpha }=2\hat{\alpha }\sigma_{1}^{2}-2\left(1-\hat{\alpha }\right)\sigma_{2}^{2}=0, \end{array}$$

from which


(1) $$\begin{array}{l} \hat{\alpha }=\frac{\sigma_{2}^{2}}{\sigma_{1}^{2}+\sigma_{2}^{2}} \end{array}$$

and the optimal variance will be:


$$\begin{array}{l} \sigma_{B}^{2}\left(\hat{\alpha }\right)=\frac{\sigma_{1}^{2}\sigma_{2}^{4}}{{\left(\sigma_{1}^{2}+\sigma_{2}^{2}\right)}^{2}}+\frac{\sigma_{2}^{2}\sigma_{1}^{4}}{{\left(\sigma_{1}^{2}+\sigma_{2}^{2}\right)}^{2}}=\frac{\sigma_{1}^{2}\sigma_{2}^{2}\left(\sigma_{1}^{2}+\sigma_{2}^{2}\right)}{{\left(\sigma_{1}^{2}+\sigma_{2}^{2}\right)}^{2}} \end{array}$$

(2) $$\begin{array}{l} =\frac{\sigma_{1}^{2}\sigma_{2}^{2}}{\sigma_{1}^{2}+\sigma_{2}^{2}} \end{array}$$

**Figure 1:**
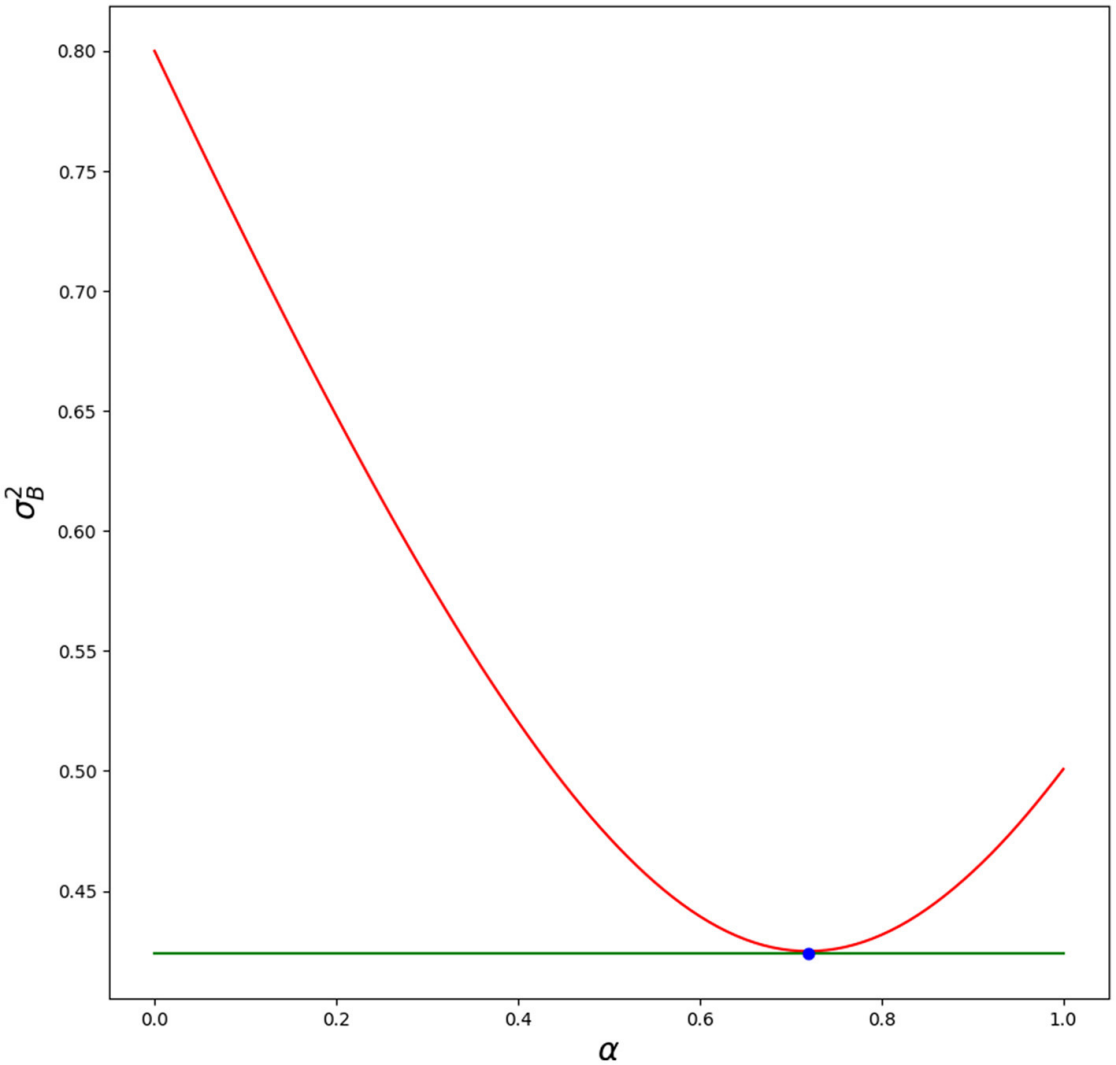
Red line—variance for different α. Green line—optimal variance $\sigma_{B}^{2}$. Blue dot—optimal α with its value $\sigma_{B}^{2}\left(\hat{\alpha }\right)$. Python script is in Appendix 1.

In Figure 1, we show how the variance is changing for different blending parameters α. Script is in Appendix 1. Blue dot—optimal value of blending parameter α matches simulation (minimal value for variance).

## Blending three independent models

Now, we consider three independent models


$$\begin{array}{l} {ŷ}_{1,j}=y_{j}+r_{1,j}\\ {ŷ}_{2,j}=y_{j}+r_{2,j},\\ {ŷ}_{3,j}=y_{j}+r_{3,j}, \end{array}$$

where $R_{1}\sim N\left(0,\sigma_{1}^{2}\right)$, $R_{2}\sim N\left(0,\sigma_{2}^{2}\right)$, and $R_{3}\sim N\left(0,\sigma_{3}^{2}\right)$.

Here, we blend optimally the first two models from the previous section:


$$\begin{array}{l} {ŷ}_{4,j}=y_{j}+\hat{\alpha }r_{1,j}+\left(1-\hat{\alpha }\right)r_{2,j}=y_{j}+r_{4,j}, \end{array}$$

where $R_{4}\sim N\left(0,\frac{\sigma_{1}^{2}\sigma_{2}^{2}}{\sigma_{1}^{2}+\sigma_{2}^{2}}\right)$ and then we find the blending parameter $\hat{\beta }$ for ŷ_3,*j*_ and ŷ_4,*j*_ such that


(3) $$\begin{array}{l} {ŷ}_{B,j}=y_{j}+\hat{\beta }r_{3,j}+\left(1-\hat{\beta }\right)r_{4,j}. \end{array}$$

Based on the Equation (1), we get


$$\begin{array}{l} \hat{\beta }=\frac{\frac{\sigma_{1}^{2}\sigma_{2}^{2}}{\sigma_{1}^{2}+\sigma_{2}^{2}}}{\sigma_{3}^{2}+\frac{\sigma_{1}^{2}\sigma_{2}^{2}}{\sigma_{1}^{2}+\sigma_{2}^{2}}}=\frac{\sigma_{1}^{2}\sigma_{2}^{2}}{\sigma_{1}^{2}\sigma_{2}^{2}+\sigma_{1}^{2}\sigma_{3}^{2}+\sigma_{2}^{2}\sigma_{3}^{2}}. \end{array}$$

Plugging this back into the Equation (3), we get


$$\begin{array}{l} {ŷ}_{B,j}=y_{j}+\frac{\sigma_{1}^{2}\sigma_{2}^{2}}{\sigma_{1}^{2}\sigma_{2}^{2}+\sigma_{1}^{2}\sigma_{3}^{2}+\sigma_{2}^{2}\sigma_{3}^{2}}r_{3,j}\\ +\left(1-\frac{\sigma_{1}^{2}\sigma_{2}^{2}}{\sigma_{1}^{2}\sigma_{2}^{2}+\sigma_{1}^{2}\sigma_{3}^{2}+\sigma_{2}^{2}\sigma_{3}^{2}}\right)r_{4,j} \end{array}$$

$$\begin{array}{l} {ŷ}_{B,j}=y_{j}+\frac{\sigma_{1}^{2}\sigma_{2}^{2}}{\sigma_{1}^{2}\sigma_{2}^{2}+\sigma_{1}^{2}\sigma_{3}^{2}+\sigma_{2}^{2}\sigma_{3}^{2}}r_{3,j}\\ +\frac{\left(\sigma_{1}^{2}+\sigma_{2}^{2}\right)\sigma_{3}^{2}}{\sigma_{1}^{2}\sigma_{2}^{2}+\sigma_{1}^{2}\sigma_{3}^{2}+\sigma_{2}^{2}\sigma_{3}^{2}}\left(\hat{\alpha }r_{1,j}+\left(1-\hat{\alpha }\right)r_{2,j}\right) \end{array}$$

$$\begin{array}{l} {ŷ}_{B,j}=y_{j}+\frac{\sigma_{1}^{2}\sigma_{2}^{2}}{\sigma_{1}^{2}\sigma_{2}^{2}+\sigma_{1}^{2}\sigma_{3}^{2}+\sigma_{2}^{2}\sigma_{3}^{2}}r_{3,j}\\ +\frac{\left(\sigma_{1}^{2}+\sigma_{2}^{2}\right)\sigma_{3}^{2}}{\sigma_{1}^{2}\sigma_{2}^{2}+\sigma_{1}^{2}\sigma_{3}^{2}+\sigma_{2}^{2}\sigma_{3}^{2}}\left(\frac{\sigma_{2}^{2}}{\sigma_{1}^{2}+\sigma_{2}^{2}}r_{1,j}+\left(1-\frac{\sigma_{2}^{2}}{\sigma_{1}^{2}+\sigma_{2}^{2}}\right)r_{2,j}\right) \end{array}$$

$$\begin{array}{l} {ŷ}_{B,j}=y_{j}+\frac{\sigma_{1}^{2}\sigma_{2}^{2}}{\sigma_{1}^{2}\sigma_{2}^{2}+\sigma_{1}^{2}\sigma_{3}^{2}+\sigma_{2}^{2}\sigma_{3}^{2}}r_{3,j}\\ +\frac{\sigma_{2}^{2}\sigma_{3}^{2}}{\sigma_{1}^{2}\sigma_{2}^{2}+\sigma_{1}^{2}\sigma_{3}^{2}+\sigma_{2}^{2}\sigma_{3}^{2}}r_{1,j}+\frac{\sigma_{1}^{2}\sigma_{3}^{2}}{\sigma_{1}^{2}\sigma_{2}^{2}+\sigma_{1}^{2}\sigma_{3}^{2}+\sigma_{2}^{2}\sigma_{3}^{2}}r_{2,j}, \end{array}$$

which is symmetrical, meaning model combination order is irrelevant. Finally for ${\hat{\alpha }}_{1}$, ${\hat{\alpha }}_{2}$, ${\hat{\alpha }}_{3}$, we get:


$$\begin{array}{l} {\hat{\alpha }}_{1}=\frac{\sigma_{2}^{2}\sigma_{3}^{2}}{\sigma_{1}^{2}\sigma_{2}^{2}+\sigma_{1}^{2}\sigma_{3}^{2}+\sigma_{2}^{2}\sigma_{3}^{2}}\\ {\hat{\alpha }}_{2}=\frac{\sigma_{1}^{2}\sigma_{3}^{2}}{\sigma_{1}^{2}\sigma_{2}^{2}+\sigma_{1}^{2}\sigma_{3}^{2}+\sigma_{2}^{2}\sigma_{3}^{2}}\\ {\hat{\alpha }}_{3}=\frac{\sigma_{1}^{2}\sigma_{2}^{2}}{\sigma_{1}^{2}\sigma_{2}^{2}+\sigma_{1}^{2}\sigma_{3}^{2}+\sigma_{2}^{2}\sigma_{3}^{2}}. \end{array}$$

Combining the second and third models first and then combining the result with the first model would lead to the same optimal blending parameters. The order of the combination is inconsequential. Additionally, for the final variance, we get from Equation (2)


$$\begin{array}{l} \sigma_{B}^{2}\left(\hat{\alpha }\right)=\frac{\sigma_{3}^{2}\frac{\sigma_{1}^{2}\sigma_{2}^{2}}{\sigma_{1}^{2}+\sigma_{2}^{2}}}{\sigma_{3}^{2}+\frac{\sigma_{1}^{2}\sigma_{2}^{2}}{\sigma_{1}^{2}+\sigma_{2}^{2}}}=\frac{\sigma_{1}^{2}\sigma_{2}^{2}\sigma_{3}^{2}}{\sigma_{1}^{2}\sigma_{2}^{2}+\sigma_{1}^{2}\sigma_{3}^{2}+\sigma_{2}^{2}\sigma_{3}^{2}}. \end{array}$$

**Figure 2:**
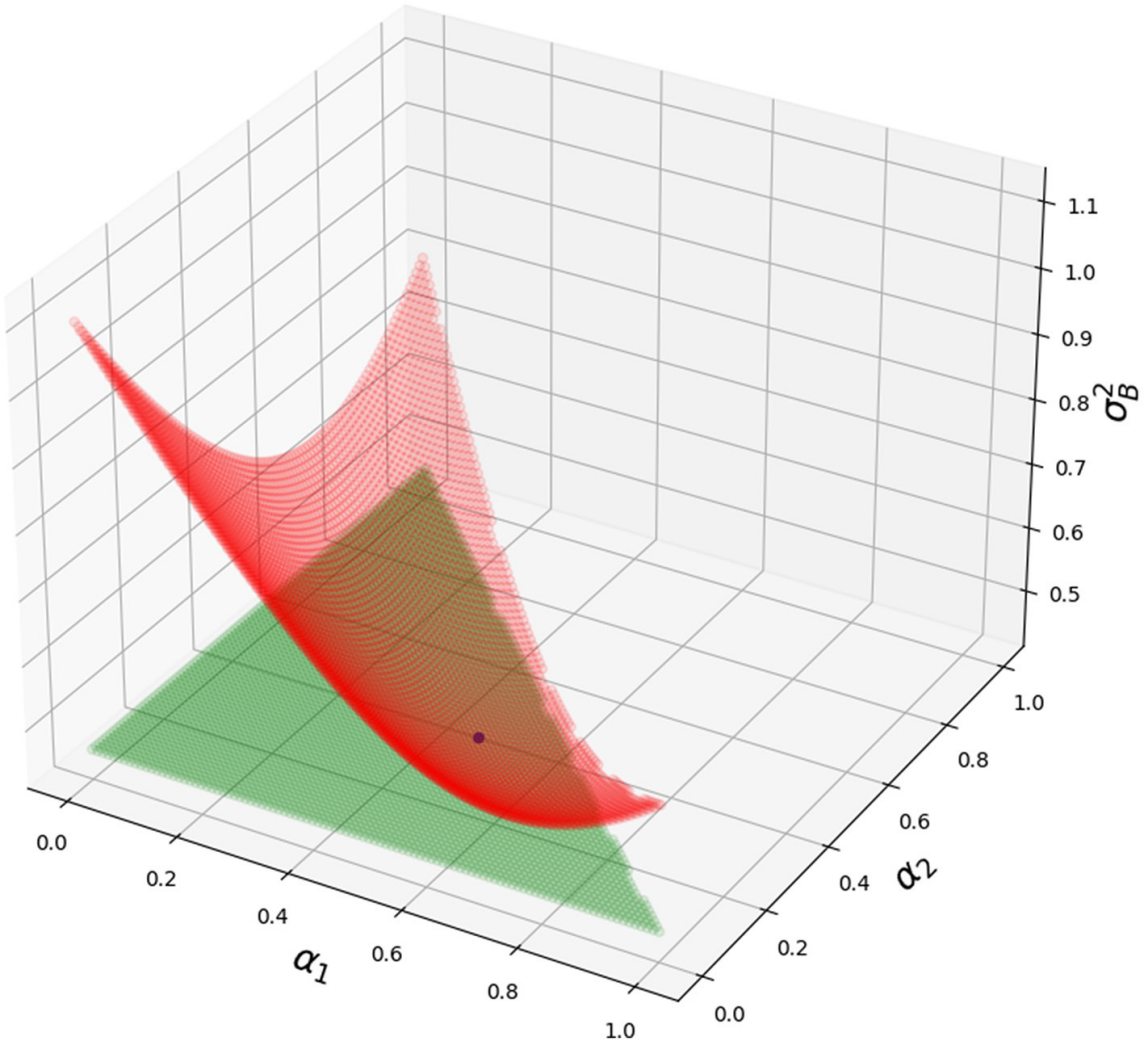
Grid consisting of red dots—variance for different α_1_ and α_2_. Grid consisting of green dots—optimal variance $\sigma_{B}^{2}$. Blue dot—optimal α_1_ and α_2_ and 1 − α_1_ − α_2_ with its value $\sigma_{B}^{2}\left(\hat{\alpha }\right)$. Python script is in Appendix 2.

In Figure 2, we show how variance is changing for different blending parameters α_1_ and α_2_ and α_3_ = 1 − α_1_ − α_2_. The script is in Appendix 2. Blue dot—the optimal value of blending parameters (α_1_, α_2_, 1 − α_1_ − α_2_) matches the simulation (minimal value for variance).

## Blending *N* independent models

Now that we have formulas for two and three different models, we prove formulas for *N* independent models with normal distributions:


$$\begin{array}{l} {ŷ}_{k,j}=y_{j}+r_{k,j}, \end{array}$$

where $R_{k}\sim N\left(0,\sigma_{k}^{2}\right)$. We combine these models as follows:


$$\begin{array}{l} {ŷ}_{B,j}=\overset{N}{\underset{k=1}{\sum }}\alpha_{k}{ŷ}_{k,j}, \end{array}$$

where $\overset{N}{\underset{k=1}{\sum }}\alpha_{k}=1$ and α_*k*_ > 0 for *k* ∈ [1, *N*].

First, we show model independence is still needed.

**Lemma 1**. *Having *N* independent models with normal distributions $R_{k}\sim N\left(0,\sigma_{k}^{2}\right)$ for *k* ∈ [1, *N*], when combined as $r_{B,j}=\overset{N}{\underset{k=1}{\sum }}\alpha_{k}r_{k,j}$, variance of *R*_*B*_ is $\sigma_{B}^{2}=\overset{N}{\underset{k=1}{\sum }}\alpha_{k}^{2}\sigma_{k}^{2}$*.

*Proof*.


$$\begin{array}{l} \sigma_{B}^{2}=E\left({\left(\overset{N}{\underset{k=1}{\sum }}\alpha_{k}r_{k,j}\right)}^{2}\right)\\ =\frac{1}{M}\left(\overset{N}{\underset{k=1}{\sum }}\overset{M}{\underset{j=1}{\sum }}\alpha_{k}^{2}r_{k,j}^{2}+2\overset{N}{\underset{k=1}{\sum }}\overset{N}{\underset{\begin{array}{c} l=1\\ l\neq k \end{array}}{\sum }}\overset{M}{\underset{j=1}{\sum }}\alpha_{k}\alpha_{l}r_{k,j}r_{l,j}\right)\\ =\overset{N}{\underset{k=1}{\sum }}\alpha_{k}^{2}\frac{1}{M}\overset{M}{\underset{j=1}{\sum }}r_{k,j}^{2}+2\overset{N}{\underset{k=1}{\sum }}\overset{N}{\underset{\begin{array}{c} l=1\\ l\neq k \end{array}}{\sum }}\alpha_{k}\alpha_{l}\frac{1}{M}\overset{M}{\underset{j=1}{\sum }}r_{k,j}r_{l,j} \end{array}$$

As models are independent (covariance $\frac{1}{M}\overset{M}{\underset{j=1}{\sum }}r_{k,j}r_{l,j}=0$ is zero for *l* ≠ *k*), we get


$$\begin{array}{l} \sigma_{B}^{2}=\overset{N}{\underset{k=1}{\sum }}\alpha_{k}^{2}\frac{1}{M}\overset{M}{\underset{j=1}{\sum }}r_{k,j}^{2}=\overset{N}{\underset{k=1}{\sum }}\alpha_{k}^{2}\sigma_{k}^{2}, \end{array}$$

which ends the proof.

**Theorem 2**. *Having *N* independent models with normal distributions $R_{k}\sim N\left(0,\sigma_{k}^{2}\right)$ for *k* ∈ [1, *N*], we get an optimal blend with parameters*


$$\begin{array}{l} \hat{\alpha_{k}}=\frac{\prod_{\begin{array}{c} j=1\\ j\neq k \end{array}}^{N}\sigma_{j}^{2}}{\sum_{i=1}^{N}\prod_{\begin{array}{c} j=1\\ j\neq i \end{array}}^{N}\sigma_{j}^{2}}, \end{array}$$

*and these independent models form normal distribution $N\left(0,\sigma_{B}^{2}\right)$, which has variance*


$$\begin{array}{l} \sigma_{B}^{2}=\frac{\prod_{j=1}^{N}\sigma_{j}^{2}}{\sum_{i=1}^{N}\prod_{\begin{array}{c} j=1\\ j\neq i \end{array}}^{N}\sigma_{j}^{2}}. \end{array}$$

*Proof*. For *N* = 2, we have shown it in Section 2. Now we use induction, if it is true for *N*, then it is true also for *N* + 1.

**Remark**. *We have shown this also for three models in Section 3, but as for induction it is not needed, Section 3 is only a motivational section for how to derive final formulas for *N* models*.

We combine two normal distributions $N\left(0,\frac{\prod_{j=1}^{N}\sigma_{j}^{2}}{\sum_{i=1}^{N}\prod_{\begin{array}{c} j=1\\ j\neq i \end{array}}^{N}\sigma_{j}^{2}}\right)$ (assuming it is true for *N*) and $N\left(0,\sigma_{N+1}^{2}\right)$. From Equation (2), (lemma 1 is incorporated in this equation) we get


$$\begin{array}{l} \sigma_{B}^{2}=\frac{\frac{\prod_{j=1}^{N}\sigma_{j}^{2}}{\sum_{i=1}^{N}\prod_{\begin{array}{c} j=1\\ j\neq i \end{array}}^{N}\sigma_{j}^{2}}\sigma_{N+1}^{2}}{\frac{\prod_{j=1}^{N}\sigma_{j}^{2}}{\sum_{i=1}^{N}\prod_{\begin{array}{c} j=1\\ j\neq i \end{array}}^{N}\sigma_{j}^{2}}+\sigma_{N+1}^{2}}\\ =\frac{\sigma_{N+1}^{2}\prod_{j=1}^{N}\sigma_{j}^{2}}{\prod_{j=1}^{N}\sigma_{j}^{2}+\sigma_{N+1}^{2}\sum_{i=1}^{N}\prod_{\begin{array}{c} j=1\\ j\neq i \end{array}}^{N}\sigma_{j}^{2}}\\ =\frac{\prod_{j=1}^{N+1}\sigma_{j}^{2}}{\sum_{i=1}^{N+1}\prod_{\begin{array}{c} j=1\\ j\neq i \end{array}}^{N+1}\sigma_{j}^{2}} \end{array}$$

and hence, we have shown optimal variance is valid for *N* + 1. Now we must show the same for the optimal coefficients. From Equation (1), we get


$$\begin{array}{l} \hat{\alpha }=\frac{\sigma_{N+1}^{2}}{\frac{\prod_{j=1}^{N}\sigma_{j}^{2}}{\sum_{i=1}^{N}\prod_{\begin{array}{c} j=1\\ j\neq i \end{array}}^{N}\sigma_{j}^{2}}+\sigma_{N+1}^{2}}=\frac{\sigma_{N+1}^{2}\sum_{i=1}^{N}\prod_{\begin{array}{c} j=1\\ j\neq i \end{array}}^{N}\sigma_{j}^{2}}{\sum_{i=1}^{N+1}\prod_{\begin{array}{c} j=1\\ j\neq i \end{array}}^{N+1}\sigma_{j}^{2}} \end{array}$$

and hence,


$$\begin{array}{l} {\hat{\alpha }}_{N+1}=1-\hat{\alpha }\\ =1-\frac{\sigma_{N+1}^{2}\sum_{i=1}^{N}\prod_{\begin{array}{c} j=1\\ j\neq i \end{array}}^{N}\sigma_{j}^{2}}{\sum_{i=1}^{N+1}\prod_{\begin{array}{c} j=1\\ j\neq i \end{array}}^{N+1}\sigma_{j}^{2}}\\ =\frac{\sum_{i=1}^{N+1}\prod_{\begin{array}{c} j=1\\ j\neq i \end{array}}^{N+1}\sigma_{j}^{2}-\sigma_{N+1}^{2}\sum_{i=1}^{N}\prod_{\begin{array}{c} j=1\\ j\neq i \end{array}}^{N}\sigma_{j}^{2}}{\sum_{i=1}^{N+1}\prod_{\begin{array}{c} j=1\\ j\neq i \end{array}}^{N+1}\sigma_{j}^{2}}\\ =\frac{\prod_{\begin{array}{c} j=1\\ j\neq N+1 \end{array}}^{N+1}\sigma_{j}^{2}+\sum_{i=1}^{N}\prod_{\begin{array}{c} j=1\\ j\neq i \end{array}}^{N+1}\sigma_{j}^{2}-\sigma_{N+1}^{2}\sum_{i=1}^{N}\prod_{\begin{array}{c} j=1\\ j\neq i \end{array}}^{N}\sigma_{j}^{2}}{\sum_{i=1}^{N+1}\prod_{\begin{array}{c} j=1\\ j\neq i \end{array}}^{N+1}\sigma_{j}^{2}}\\ =\frac{\prod_{\begin{array}{c} j=1\\ j\neq N+1 \end{array}}^{N+1}\sigma_{j}^{2}}{\sum_{i=1}^{N+1}\prod_{\begin{array}{c} j=1\\ j\neq i \end{array}}^{N+1}\sigma_{j}^{2}}, \end{array}$$

which proves ${\hat{\alpha }}_{N+1}$. Finally to show the same for $\hat{\alpha_{k}}$ for *k* ∈ [1, *N*]:


$$\begin{array}{l} \hat{\alpha_{k}}=\hat{\alpha }\frac{\prod_{\begin{array}{c} j=1\\ j\neq k \end{array}}^{N}\sigma_{j}^{2}}{\sum_{i=1}^{N}\prod_{\begin{array}{c} j=1\\ j\neq i \end{array}}^{N}\sigma_{j}^{2}}\\ =\frac{\sigma_{N+1}^{2}\sum_{i=1}^{N}\prod_{\begin{array}{c} j=1\\ j\neq i \end{array}}^{N}\sigma_{j}^{2}}{\sum_{i=1}^{N+1}\prod_{\begin{array}{c} j=1\\ j\neq i \end{array}}^{N+1}\sigma_{j}^{2}}\frac{\prod_{\begin{array}{c} j=1\\ j\neq k \end{array}}^{N}\sigma_{j}^{2}}{\sum_{i=1}^{N}\prod_{\begin{array}{c} j=1\\ j\neq i \end{array}}^{N}\sigma_{j}^{2}}\\ =\frac{\sigma_{N+1}^{2}}{\sum_{i=1}^{N+1}\prod_{\begin{array}{c} j=1\\ j\neq i \end{array}}^{N+1}\sigma_{j}^{2}}\overset{N}{\underset{\begin{array}{c} j=1\\ j\neq k \end{array}}{\prod }}\sigma_{j}^{2}\\ =\frac{\prod_{\begin{array}{c} j=1\\ j\neq k \end{array}}^{N+1}\sigma_{j}^{2}}{\sum_{i=1}^{N+1}\prod_{\begin{array}{c} j=1\\ j\neq i \end{array}}^{N+1}\sigma_{j}^{2}}, \end{array}$$

which ends the proof.

## Going to infinity

If we can generate infinite independent models with distributions $R_{i}\sim N\left(0,\sigma^{2}\right)$ (same variance), the final variance will be


$$\begin{array}{l} \sigma_{B}^{2}=\underset{N\to +\infty }{\lim}\frac{\prod_{j=1}^{N}\sigma^{2}}{\sum_{i=1}^{N}\prod_{\begin{array}{c} j=1\\ j\neq i \end{array}}^{N}\sigma^{2}}=\underset{N\to +\infty }{\lim}\frac{\sigma^{2N}}{N\sigma^{2\left(N-1\right)}}\\ =\underset{N\to +\infty }{\lim}\frac{\sigma^{2}}{N}=0, \end{array}$$

which means we can combine all these models to get a perfect prediction with no errors. Naturally, creating an infinite amount of independent models (with covariances zero) is a difficult if not impossible task in real applications.

**Theorem 3**. *Having *N* independent models with normal distributions $R_{k}\sim N\left(0,\sigma_{k}^{2}\right)$ for *k* ∈ [1, *N*] and their variances $\sigma_{k}^{2}\leq \sigma_{M}^{2}$, where $\sigma_{M}^{2}$ is their maximum variance, combining them optimally with coefficients from the theorem 2, their combined variance is $\sigma_{B}^{2}\leq \frac{\sigma_{M}^{2}}{N}$*.

*Proof*. We use induction again. For *N* = 2, we get


$$\begin{array}{l} \sigma_{B}^{2}=\frac{\sigma_{1}^{2}\sigma_{2}^{2}}{\sigma_{1}^{2}+\sigma_{2}^{2}}\leq \frac{\sigma_{M}^{2}}{2} \end{array}$$

This is true as


$$\begin{array}{l} \sigma_{1}^{2}\sigma_{2}^{2}+\sigma_{1}^{2}\sigma_{2}^{2}\leq \sigma_{M}^{2}\sigma_{1}^{2}+\sigma_{M}^{2}\sigma_{2}^{2}, \end{array}$$

because


$$\begin{array}{l} \sigma_{1}^{2}\sigma_{2}^{2}\leq \sigma_{M}^{2}\sigma_{1}^{2} \end{array}$$

and


$$\begin{array}{l} \sigma_{1}^{2}\sigma_{2}^{2}\leq \sigma_{M}^{2}\sigma_{2}^{2}. \end{array}$$

Now if it is true for *N*, then it is true also for *N* + 1. If


$$\begin{array}{l} \sigma_{B,N}^{2}\leq \frac{\sigma_{M}^{2}}{N}, \end{array}$$

then


$$\begin{array}{l} \sigma_{B,N+1}^{2}\leq \frac{\sigma_{M}^{2}}{N+1}. \end{array}$$

That is true as


$$\begin{array}{l} \sigma_{B,N+1}^{2}=\frac{\sigma_{B,N}^{2}\sigma_{N+1}^{2}}{\sigma_{B,N}^{2}+\sigma_{N+1}^{2}}\leq \frac{\sigma_{M}^{2}}{N+1}, \end{array}$$

because


$$\begin{array}{l} N\sigma_{B,N}^{2}\sigma_{N+1}^{2}+\sigma_{B,N}^{2}\sigma_{N+1}^{2}\leq \sigma_{M}^{2}\left(\sigma_{B,N}^{2}+\sigma_{N+1}^{2}\right) \end{array}$$

as both - this


$$\begin{array}{l} N\sigma_{B,N}^{2}\sigma_{N+1}^{2}\leq \sigma_{M}^{2}\sigma_{N+1}^{2} \end{array}$$

and this


$$\begin{array}{l} \sigma_{B,N}^{2}\sigma_{N+1}^{2}\leq \sigma_{M}^{2}\sigma_{B,N}^{2} \end{array}$$

are true, which ends the proof.

This proof means, that if we combine infinite independent models with distributions $R_{i}\sim N\left(0,\sigma_{i}^{2}\right)$, where variance $\sigma_{i}^{2}\leq \sigma_{M}^{2}$, we get variance:


$$\begin{array}{l} \sigma_{B}^{2}=\underset{N\to +\infty }{\lim}\frac{\prod_{j=1}^{N}\sigma_{j}^{2}}{\sum_{i=1}^{N}\prod_{\begin{array}{c} j=1\\ j\neq i \end{array}}^{N}\sigma_{j}^{2}}\leq \underset{N\to +\infty }{\lim}\frac{\sigma_{M}^{2}}{N}=0. \end{array}$$

Combining infinite independent models with bounded variances from above leads to perfect prediction with variance zero.

It can be shown the same way as in Theorem 3 that combined variance is bounded also from below (as the proof is almost identical we avoid it here). If all distributions $R_{k}\sim N\left(0,\sigma_{k}^{2}\right)$ for *k* ∈ [1, *N*] have their variance in interval $\sigma_{k}^{2}\in \left[\sigma_{min}^{2},\sigma_{max}^{2}\right]$ for *k* ∈ [1, *N*], then their combined variance will be in interval $\sigma_{B}^{2}\in \left[\frac{\sigma_{min}^{2}}{N},\frac{\sigma_{max}^{2}}{N}\right]$.

## Similar conclusion with machine learning with counts

When it comes to using only counts (feature says yes or no only) in machine learning for predictions, as it is shown in Taraba (2021) (see section 7) on a nine-features example, we can come to the same conclusion as in the previous chapter that an infinite amount of features can lead to perfect prediction with no error. While the previous approach is statistical, machine learning with counts uses Pascal's triangle and binomial raised to infinity to show this. We use the binomial expansion


1=(p+(1-p))n=∑i=0n(ni)pn-i(1-p)i,

where *p* is the probability of features to be correct. As we want to have an odd amount of features to be able to make a decision purely on the counts (feature says yes or no), we will replace *n* with 2*k* + 1


1=(p+(1-p))2k+1=∑i=02k+1(2k+1i)p2k+1-i(1-p)i.

This can be split into two parts, one with probability when the majority of features are correct *P*_*correct*_ and one with probability when the majority of features are incorrect:


$$\begin{array}{l} 1={\left(p+\left(1-p\right)\right)}^{2k+1}=P_{correct}+P_{incorrect}, \end{array}$$

where


Pcorrect=∑i=0k(2k+1i)p2k+1-i(1-p)i

and


Pincorrect=∑i=k+12k+1(2k+1i)p2k+1-i(1-p)i.

To show that an infinite amount of features can lead to perfect prediction, we have to show that *P*_*correct*_ with the majority of features correct (at least *k* + 1 of them correct) goes to 1 for all *p* ∈ (0.5, 1]


limk→∞∑i=0k(2k+1i)p2k+1-i(1-p)i=1

We start by showing the simpler case first and that is when *p* = 0.5 then *P*_*correct*_ and *P*_*incorrect*_ are equal and *P*_*correct*_ = *P*_*incorrect*_ = 0.5. To show this, we can write


Pcorrect,p=0.5=∑i=0k(2k+1i)0.52k+1=0.52k+1∑i=0k(2k+1i)

and


Pincorrect,p=0.5=∑i=k+12k+1(2k+1i)0.52k+1=0.52k+1∑i=k+12k+1(2k+1i)

and those are equal as ∑i=0k(2k+1i)=∑i=k+12k+1(2k+1i), because (2k+1i)=(2k+12k+1-i) for *i* ∈ {0, 1, …, *k*}. As *P*_*correct,p* = 0.5_ = *P*_*incorrect,p* = 0.5_ and their sum is 1 it follows that


$$\begin{array}{l} 1=P_{correct,p=0.5}+P_{incorrect,p=0.5}=2P_{correct,p=0.5} \end{array}$$

and hence,


$$\begin{array}{l} P_{correct,p=0.5}=P_{incorrect,p=0.5}=0.5. \end{array}$$

Now that we have shown what happens when *p* = 0.5, we show the main limit theorem for *p* ∈ (0.5, 1].

**Theorem 4**. *limk→∞∑i=0k(2k+1i)p2k+1-i(1-p)i=1 for all* *p* ∈ (0.5, 1].

*Proof*. To show that limk→∞∑i=0k(2k+1i)p2k+1-i(1-p)i=1 for all *p* ∈ (0.5, 1], we will show instead that limk→∞∑i=k+12k+1(2k+1i)p2k+1-i(1-p)i=0 and as their sum is 1, limk→∞∑i=0k(2k+1i)p2k+1-i(1-p)i=1 will follow.

First, we can rewrite ∑i=k+12k+1(2k+1i)p2k+1-i(1-p)i as


∑i=k+12k+1(2k+1i)p2k+1-i(1-p)i=∑i=0k(2k+1k+1+i)p2k+1-(k+1+i)(1-p)k+1+i.

It is obvious that the numbers in the pascal triangle are decreasing when starting after the middle:


(2k+1k+1+i+1)(2k+1k+1+i)=(2k+1)!(k+2+i)!(k-i-1)!(2k+1)!(k+1+i)!(k-i)!=k-ik+i+2<1

for *i* ∈ 0, 1, …, *k* − 1, and hence, we can write


∑i=0k(2k+1k+1+i)p2k+1-(k+1+i)(1-p)k+1+i<(2k+1k+1)pk(1-p)k+1∑i=0k(1-pp)i.

As *p* is in *p* ∈ (0.5, 1], then $\frac{1-p}{p}\in \left[0,1\right)$, and hence, we can write


(2k+1k+1)pk(1-p)k+1∑i=0k(1-pp)i=(2k+1k+1)pk(1-p)k+11-(1-pp)k+11-(1-pp).

With that we can finally look at the original limit and write


limk→∞∑i=k+12k+1(2k+1i)p2k+1-i(1-p)i≤limk→∞(2k+1k+1)pk(1-p)k+11-(1-pp)k+11-(1-pp).

As $\underset{k\to \infty }{\lim}{\left(\frac{1-p}{p}\right)}^{k+1}=0$ for *p* ∈ (0.5, 1] as $\frac{1-p}{p}\in \left[0,1\right)$, we can write


limk→∞∑i=k+12k+1(2k+1i)p2k+1-i(1-p)i≤11-(1-pp)limk→∞(2k+1k+1)pk(1-p)k+1=p2p-1limk→∞(2k+1k+1)pk(1-p)k+1.

Now we look at limk→∞(2k+1k+1)pk(1-p)k+1. We can take a member (2(k+r)+1(k+r)+1)p(k+r)(1-p)(k+r)+1 and compare it with *r*′ = *r* + 1 follower (2(k+r+1)+1(k+r+1)+1)p(k+r+1)(1-p)(k+r+1)+1 by division


(2(k+r+1)+1(k+r+1)+1)p(k+r+1)(1-p)(k+r+1)+1(2(k+r)+1(k+r)+1)p(k+r)(1-p)(k+r)+1=(2(k+r)+3)!(k+r+2)!(k+r+1)!(2(k+r)+1)!(k+r+1)!(k+r)!p(1-p)==(2(k+r)+3)(2(k+r)+2)(k+r+2)(k+r+1)p(1-p)<4p(1-p)<1,

for all *r* ≥ 0 and *p* ∈ (0.5, 1] as *p*(1 − *p*) ∈ [0, 0.25). This means we are multiplying a finite number (2k+1k+1)pk(1-p)k+1, which is in interval [0, 1], infinitely many times with a number larger or equal than 0 and smaller than 1, hence


limk→∞(2k+1k+1)pk(1-p)k+1=0

and hence, the original limit


limk→∞∑i=k+12k+1(2k+1i)p2k+1-i(1-p)i≤0

as well. As ∑i=k+12k+1(2k+1i)p2k+1-i(1-p)i≥0, it follows that


limk→∞∑i=k+12k+1(2k+1i)p2k+1-i(1-p)i=0.

**Remark**. *It is worth mentioning that *p* is fixed and chosen from the interval (0.5, 1], and we are not looking at the limit of *p* → 0.5, but the limit of *k* → ∞. For *p* = 0.5, we already know that *P*_*correct,p* = 0.5_ = *P*_*incorrect,p* = 0.5_ = 0.5*.

As


limk→∞∑i=0k(2k+1i)p2k+1-i(1-p)i++limk→∞∑i=k+12k+1(2k+1i)p2k+1-i(1-p)i=1,

it follows that limk→∞∑i=0k(2k+1i)p2k+1-i(1-p)i=1, which ends the proof.

**Figure 3:**
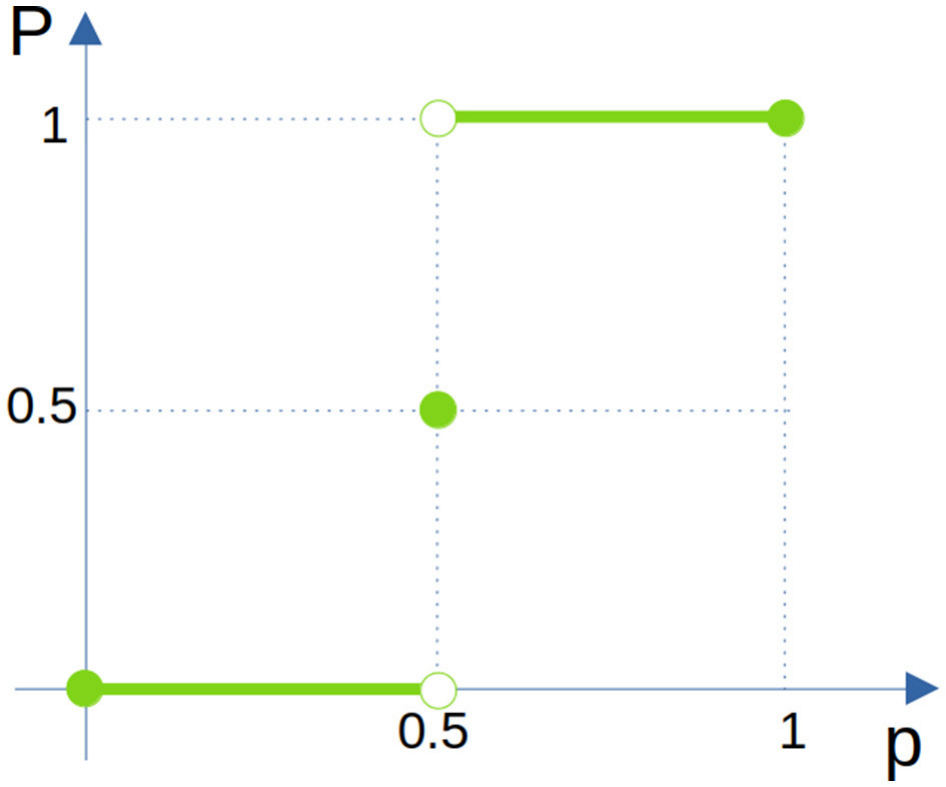
Different outcomes for *P*_*correct*_ for different *p* intervals when *k* is going to ∞.

This could be summarized for all *p* ∈ [0, 1] in Figure 3 as


$$\begin{array}{l} P_{correct}=\{\begin{array}{cc} 0 & \text{for }p\in \left[0,0.5\right)\\ 0.5 & \text{for }p=0.5\\ 1 & \text{for }p\in \left(0.5,1\right] \end{array}. \end{array}$$

**Figure 4:**
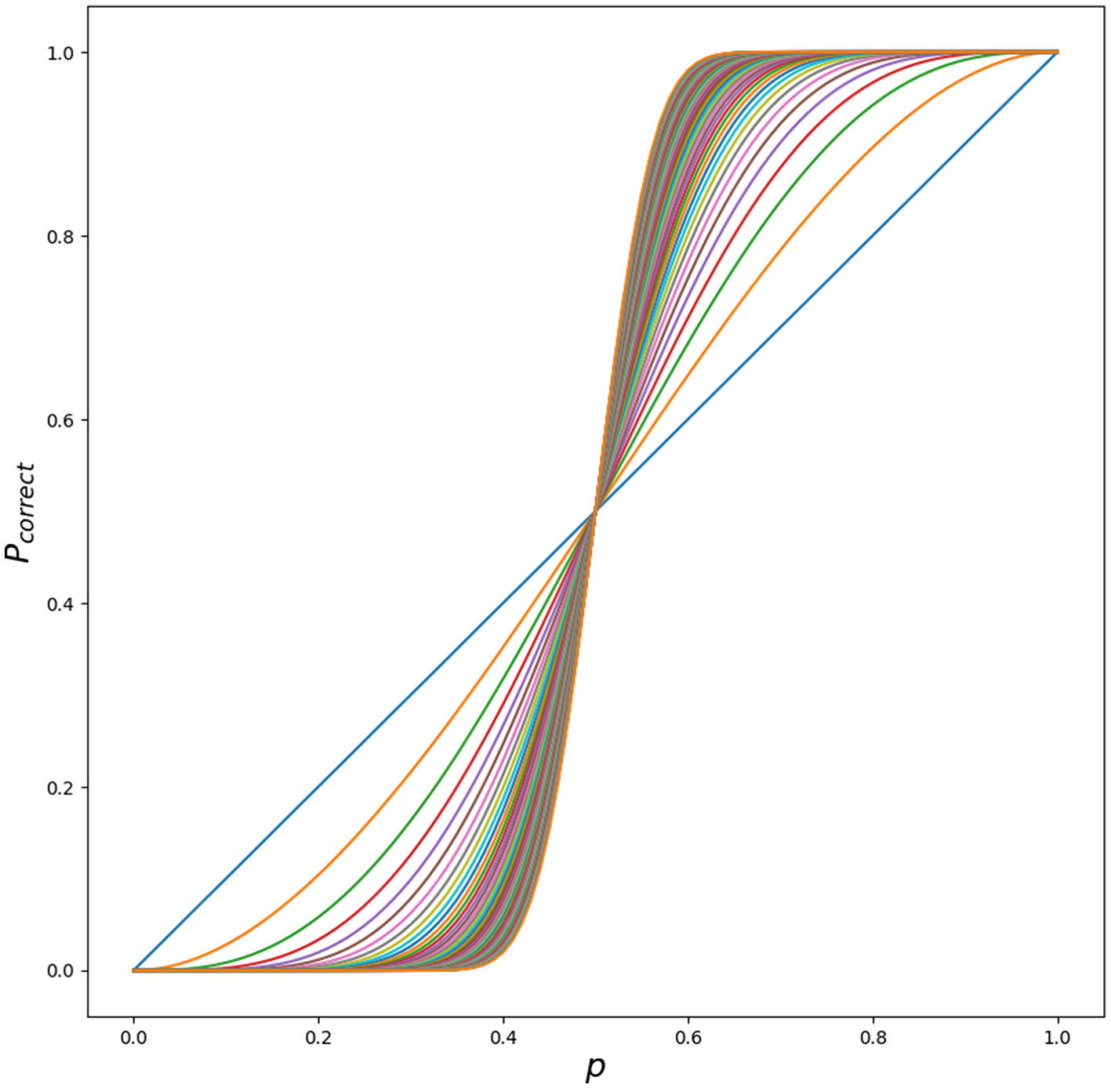
Different outcomes for *P*_*correct*_ for different *p* ∈ [0.0, 1] with increasing *k* ∈ [0, …, 51]. Python script is in Appendix 3.

This can be understood intuitively by plotting *P*_*correct*_ with increasing *k* in Figure 4.

**Figure 5:**
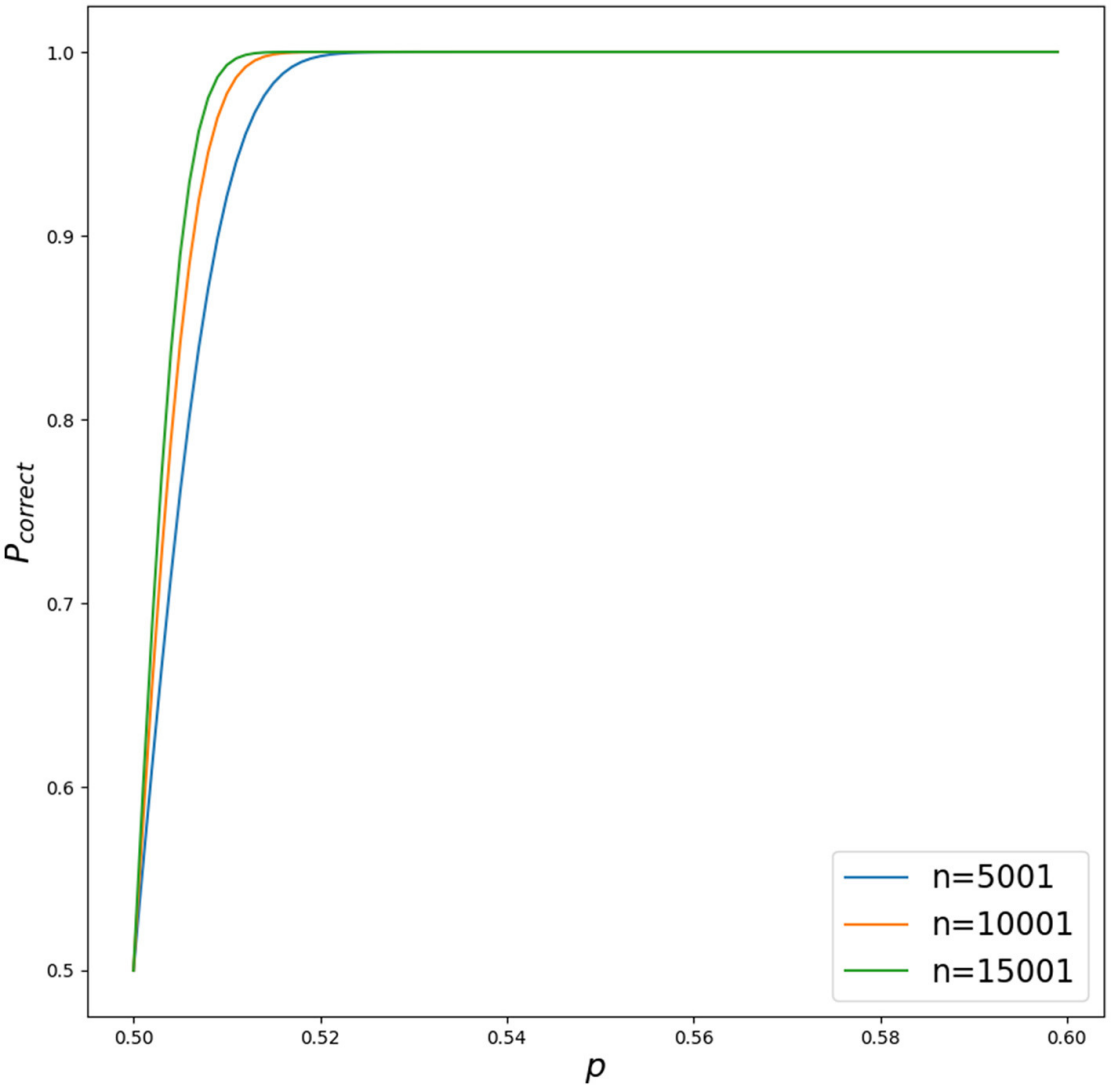
Different outcomes for *P*_*correct*_ for different *p* ∈ [0.5, 0.6] with increasing *n* for *n* ∈ {5001, 10001, 15001}. Python script is in Appendix 4.

It is worth mentioning that with weak features with *p* = 0.52, and only *n* = 15001 of them (no need to go to infinity), *P*_*correct*_ is already around 1 (see Figure 5).

As next, we show that *P*_*correct*_ increases, if any of the probability of the features increases its value $p_{i}^{\prime }>p_{i}$. For case *k* = 0 (*n* = 1), this is simple, as


$$\begin{array}{l} P_{correct}\left(new\right)=p_{1}^{\prime }>p_{1}=P_{correct}\left(old\right). \end{array}$$

For three features, it is also trivial as


$$\begin{array}{l} P_{correct}\left(new\right)=p_{1}^{\prime }p_{2}p_{3}+p_{1}^{\prime }p_{2}\left(1-p_{3}\right)+p_{1}^{\prime }\left(1-p_{2}\right)p3\\ +\left(1-p_{1}^{\prime }\right)p_{2}p_{3} \end{array}$$

as it can be re-written to $p_{1}^{\prime }$ independent part and $p_{1}^{\prime }$ dependent part


$$\begin{array}{l} P_{correct}\left(new\right)=p_{2}p_{3}+p_{1}^{\prime }\left(p_{2}\left(1-p_{3}\right)+\left(1-p_{2}\right)p_{3}\right) \end{array}$$

and from that, it immediately follows that *P*_*correct*_(*new*) > *P*_*correct*_(*old*) as $0\leq p_{1},p_{1}^{\prime },p_{2},p_{3}\leq 1$ and $p_{1}^{\prime }>p_{1}$. To show this for the general case, not only for cases *k* ∈ {0, 1} (*n* = 2*k* + 1 ∈ {1, 3}), we first write the general formula:


(4) $$\begin{array}{l} 1=\overset{1}{\underset{i_{1}=0}{\sum }}\overset{1}{\underset{i_{2}=0}{\sum }}\ldots \overset{1}{\underset{i_{2k+1}=0}{\sum }}\overset{2k+1}{\underset{j=1}{\prod }}\left(p_{j}{\left(-1\right)}^{i_{j}+1}+\left(1-i_{j}\right)\right). \end{array}$$

This can be re-written into two parts once again, *P*_*correct*_, when the majority of features say yes—are correct, and *P*_*incorrect*_, when the majority of features say no—are incorrect.


$$\begin{array}{l} P_{correct}\left(p_{1},\ldots ,p_{2k+1}\right)=\overset{1}{\underset{i_{1}=0}{\sum }}\ldots \overset{1}{\underset{i_{2k+1}=0}{\sum }}m\left(i_{1},\ldots ,i_{2k+1}\right)\\ \overset{2k+1}{\underset{j=1}{\prod }}\left(p_{j}{\left(-1\right)}^{i_{j}+1}+\left(1-i_{j}\right)\right), \end{array}$$

where


$$\begin{array}{l} m\left(i_{1},\ldots ,i_{2k+1}\right)=\{\begin{array}{cc} 0 & \text{for }\sum_{j=1}^{2k+1}i_{j}\leq k\\ 1 & \text{for }\sum_{j=1}^{2k+1}i_{j}>k \end{array}. \end{array}$$

**Theorem 5**. *Increasing the probability of one of the features $p_{1}^{\prime }>p_{1}$ increases final correct probability of all features, $P_{correct}\left(p_{1}^{\prime },p_{2},\ldots ,p_{2k+1}\right)>P_{correct}\left(p_{1},p_{2},\ldots ,p_{2k+1}\right)$*.

*Proof*. We show this for increasing the first probability, as probabilities can be re-ordered and their order does not matter for the final correct probability.

Now again, as with example *k* = 1, *n* = 3, *P*_*correct*_ can be split into $p_{1}^{\prime }$ independent and dependent part. The dependent part will only contain $p_{1}^{\prime }$ and no $\left(1-p_{1}^{\prime }\right)$ as for every $\left(1-p_{1}^{\prime }\right)$ there is also the exact same case with $p_{1}^{\prime }$, where even more features are correct and those two can be joined and hence it belongs to the independent part. Hence,


$$\begin{array}{l} P_{correct}\left(p_{1}^{\prime },p_{2},\ldots ,p_{2k+1}\right)=P_{I}+p_{1}^{\prime }P_{D}, \end{array}$$

and


$$\begin{array}{l} P_{correct}\left(p_{1},p_{2},\ldots ,p_{2k+1}\right)=P_{I}+p_{1}P_{D}, \end{array}$$

where *P*_*I*_ and *P*_*D*_ are fixed and non-negative (as all *p*_*i*_ ≥ 0 and (1 − *p*_*i*_) ≥ 0) and dependent on *p*_2_, …, *p*_2*k*+1_ and hence $P_{correct}\left(p_{1}^{\prime },p_{2},\ldots ,p_{2k+1}\right)=P_{I}+p_{1}^{\prime }P_{D}>P_{I}+p_{1}P_{D}=P_{correct}\left(p_{1},p_{2},\ldots ,p_{2k+1}\right)$ as $p_{1}^{\prime }>p_{1}$, which ends the proof.

**Theorem 6**. *Pcorrect(p1,p2,…,p2k+1)≥∑i=0k(2k+1i)pmin2k+1-i(1-pmin)i, where *p*_*min*_ =* min{*p*_1_, *p*_2_, …, *p*_2*k*+1_}.

*Proof*. This proof directly follows from using the Theorem 5 multiple times by increasing every probability one at a time from the initial value *p*_*min*_:


Pcorrect(p1,p2,…,p2k+1)≥≥Pcorrect(pmin,p2,…,p2k+1)≥≥Pcorrect(pmin,pmin,…,p2k+1)≥⋮≥Pcorrect(pmin,pmin,…,pmin)==∑i=0k(2k+1i)pmin2k+1-i(1-pmin)i,

which ends the proof.

Theorems 6 and 4 were needed in order to be able to say, that combining an infinite amount of features with their probabilities *p*_*i*_ ∈ (0.5, 1] (*min*{*p*_1_, …, *p*_2*k*+1_, …} > 0.5) will lead to perfect prediction with no error for machine learning with counts, as it was in previous Section 5 when looking at the same problem from the statistical point of view (Gaussian distributions). It is important to say once again that these separate probabilities *p*_*i*_ have to be independent, as otherwise Equation (4) would not be valid.

## Conclusion and discussion

We have derived blending coefficients for the ensemble of multiple independent prediction models with normal error distribution. This manuscript was mainly inspired by a Netflix competition, in which in the final stages of competition multiple teams joined their efforts to increase the accuracy of their final predictor and blending turned out to be essential to win the competition in a very short time during the final stage. This method was not only used in the Netflix competition but is used for other datasets in machine learning, such as MNIST and CIFAR-10 for image processing. We have also shown that having an infinite amount of independent predictors with their variances bounded from above is sufficient to achieve perfect prediction. While deep learning is very popular these days, one should not forget to include more features (going wider) when in need of improvement in accuracy.

Looking at a similar problem and more specifically machine learning with counts, where we only count how many features are for and against and make a decision based on a voting mechanism and a majority vote winner, we have shown once again, that an infinite amount of independent features will lead to perfect prediction when using only features which have >50% of accuracy.

Naturally, independent features in practice are hard to find and further the study could be made on how to convert dependent (correlated) features into independent in order to achieve as high accuracy as possible, or how to combine these dependent features together and what theoretical accuracies can be achieved. It could be also of interest how to combine features, which are not binary (for and against features), but have more than two possible outcomes.

## Data availability statement

The original contributions presented in the study are included in the article/Supplementary material, further inquiries can be directed to the corresponding author.

## Author contributions

The author confirms being the sole contributor of this work and has approved it for publication.
